# Supplementary material for: Lead ammunition residues in a hunted Australian grassland bird, the stubble quail (Coturnix pectoralis): Implications for human and wildlife health
Source: PLoS One. 2022 Apr 21;17(4):e0267401. doi: 10.1371/journal.pone.0267401 (PMC9022800; doi:10.1371/journal.pone.0267401)
Supplement: Attachment — Submitted filename: PONE-D-21-40122.pdf [file pone.0267401.s002.pdf]

PLOS ONE

Lead ammunition residues in a hunted Australian grassland bird, the stubble quail (Coturnix pectoralis): implications for human and wildlife health

--Manuscript Draft--

**Manuscript Number:**	PONE-D-21-40122
**Article Type:**	Research Article
**Full Title:**	Lead ammunition residues in a hunted Australian grassland bird, the stubble quail (Coturnix pectoralis): implications for human and wildlife health
**Short Title:**	Lead contamination and stubble quail hunting
**Corresponding Author:**	Jordan O. Hampton<br>University of Melbourne Faculty of Veterinary and Agricultural Sciences<br>Parkville, VIC AUSTRALIA
**Keywords:**	ecotoxicology; heavy metals; hunting; lead; public health; wildlife management
**Abstract:**	Scavenging and predatory wildlife can ingest lead from ammunition and become poisoned when feeding on injured, or killed but unretrieved, game animals. Humans can similarly be exposed to ammunition-derived lead when consuming wild-shot game animals. Many studies have been performed to assess the degree of lead contamination in the carcasses of game animals to inform this risk. This scrutiny has not so far extended to Australia. Stubble quail (Coturnix pectoralis) is the only native non-waterfowl bird species that can be legally hunted in Victoria, Australia, and is commonly hunted with lead shot. The aim of this study was to characterize lead contamination in harvested stubble quail. The frequency, dimensions, and number of lead fragments embedded in carcasses were assessed through use of radiography (X-ray). From these data, the average quantity of lead available to scavenging wildlife was estimated along with potential risks to human consumers. We radiographed 37 stubble quail killed by hunters using lead shot (#9, 28 g) from 12-gauge shotguns in western Victoria, Australia, in Autumn 2021. Radiographs revealed that 81% of carcasses contained embedded pellets and/or fragments. We counted 60 shotgun pellets in 24 carcasses. In 14 birds, we detected both whole pellets and lead fragments; in 10, only pellets; in 6, we found only small fragments; and in 7, we found neither pellets nor fragments. On average, 1.62 embedded pellets were detected per bird. By excising and weighing a sample of 30 shotgun pellets (all had a mass of ~0.75 grain or 48.6 mg), we calculated an average lead load of 78 mg/100 g of body mass. This was a conservative estimate, because fragments were not considered. This level of lead contamination was comparable to hunted bird species examined using similar methods in Europe. The quantity and characteristics of lead ammunition residues found suggest that predatory and scavenging wildlife feeding on Australian stubble quail will be at risk of negative health impacts: health risks are also likely to extend to some groups of human consumers.
**Order of Authors:**	Jordan O Hampton
	Heath Dunstan
	Simon D Toop
	Jason S Flesch
	Alessandro Andreotti
	Deborah J Pain
**Additional Information:**	
**Question**	**Response**
**Financial Disclosure**<br><br>Enter a financial disclosure statement that describes the sources of funding for the	The McKenzie Fellowship program of the University of Melbourne and the Victorian Game Management Authority funded this study. The funders did play a role in the preparation of the manuscript (three employees of the Game Management Authority are authors (HD, SDT and JSF)). The funders did not play a role in the analysis or

Powered by Editorial Manager® and ProduXion Manager® from Aries Systems Corporation

work included in this submission. Review the submission guidelines for detailed requirements. View published research articles from *PLOS ONE* for specific examples.<br><br>This statement is required for submission and **will appear in the published article** if the submission is accepted. Please make sure it is accurate.<br><br>**Unfunded studies**<br>Enter: *The author(s) received no specific funding for this work.*<br><br>**Funded studies**<br>Enter a statement with the following details:<br>• Initials of the authors who received each award<br>• Grant numbers awarded to each author<br>• The full name of each funder<br>• URL of each funder website<br>• Did the sponsors or funders play any role in the study design, data collection and analysis, decision to publish, or preparation of the manuscript?<br>• **NO** - Include this sentence at the end of your statement: *The funders had no role in study design, data collection and analysis, decision to publish, or preparation of the manuscript.*<br>• **YES** - Specify the role(s) played.<br><br>* typeset	decision to publish.
**Competing Interests**<br><br>Use the instructions below to enter a competing interest statement for this submission. On behalf of all authors, disclose any competing interests that could be perceived to bias this work—acknowledging all financial support and any other relevant financial or non-financial competing interests.<br><br>This statement is required for submission and **will appear in the published article** if the submission is accepted. Please make sure it is accurate and that any funding sources listed in your Funding Information	Three authors (HD, SDT and JSF) are employees of the Game Management Authority, a government agency involved with the regulation of recreational hunting activities in the state of Victoria, Australia. Their employment could not reasonably have interfered with the full and objective presentation of this research.

later in the submission form are also declared in your Financial Disclosure statement.<br><br>View published research articles from *PLOS ONE* for specific examples.<br><br>**NO authors have competing interests**<br><br>Enter: *The authors have declared that no competing interests exist.*<br><br>**Authors with competing interests**<br><br>Enter competing interest details beginning with this statement:<br><br>*I have read the journal's policy and the authors of this manuscript have the following competing interests: [insert competing interests here]*<br><br>* typeset	
**Ethics Statement**<br><br>Enter an ethics statement for this submission. This statement is required if the study involved:<br><br>• Human participants<br>• Human specimens or tissue<br>• Vertebrate animals or cephalopods<br>• Vertebrate embryos or tissues<br>• Field research<br><br>Write "N/A" if the submission does not require an ethics statement.<br><br>General guidance is provided below. Consult the submission guidelines for detailed instructions. **Make sure that all information entered here is included in the Methods section of the manuscript.**	An observational Animal Ethics Committee (AEC) licence from Murdoch University (O3103/19) supported this study.

Powered by Editorial Manager® and ProduXion Manager® from Aries Systems Corporation

**Format for specific study types**<br><br>**Human Subject Research (involving human participants and/or tissue)**<br>• Give the name of the institutional review board or ethics committee that approved the study<br>• Include the approval number and/or a statement indicating approval of this research<br>• Indicate the form of consent obtained (written/oral) or the reason that consent was not obtained (e.g. the data were analyzed anonymously)<br><br>**Animal Research (involving vertebrate animals, embryos or tissues)**<br>• Provide the name of the Institutional Animal Care and Use Committee (IACUC) or other relevant ethics board that reviewed the study protocol, and indicate whether they approved this research or granted a formal waiver of ethical approval<br>• Include an approval number if one was obtained<br>• If the study involved *non-human primates*, add *additional details* about animal welfare and steps taken to ameliorate suffering<br>• If anesthesia, euthanasia, or any kind of animal sacrifice is part of the study, include briefly which substances and/or methods were applied<br><br>**Field Research**<br><br>Include the following details if this study involves the collection of plant, animal, or other materials from a natural setting:<br>• Field permit number<br>• Name of the institution or relevant body that granted permission	
**Data Availability**<br><br>Authors are required to make all data underlying the findings described fully available, without restriction, and from the time of publication. PLOS allows rare exceptions to address legal and ethical concerns. See the PLOS Data Policy and FAQ for detailed information.	Yes - all data are fully available without restriction

A Data Availability Statement describing where the data can be found is required at submission. Your answers to this question constitute the Data Availability Statement and **will be published in the article**, if accepted.<br><br>**Important:** Stating 'data available on request from the author' is not sufficient. If your data are only available upon request, select 'No' for the first question and explain your exceptional situation in the text box.<br><br>Do the authors confirm that all data underlying the findings described in their manuscript are fully available without restriction?	
**Describe where the data may be found in full sentences. If you are copying our sample text, replace any instances of XXX with the appropriate details.**<br><br>• If the data are **held or will be held in a public repository**, include URLs, accession numbers or DOIs. If this information will only be available after acceptance, indicate this by ticking the box below. For example: *All XXX files are available from the XXX database (accession number(s) XXX, XXX.).*<br>• If the data are all contained **within the manuscript and/or Supporting Information files**, enter the following: *All relevant data are within the manuscript and its Supporting Information files.*<br>• If neither of these applies but you are able to provide **details of access elsewhere**, with or without limitations, please do so. For example:<br><br>*Data cannot be shared publicly because of [XXX]. Data are available from the XXX Institutional Data Access / Ethics Committee (contact via XXX) for researchers who meet the criteria for access to confidential data.*<br><br>*The data underlying the results presented in the study are available from (include the name of the third party*	All relevant data are within the manuscript and its Supporting Information files.

and contact information or URL).<br>• This text is appropriate if the data are owned by a third party and authors do not have permission to share the data.<br><br>* typeset	
Additional data availability information:	

Powered by Editorial Manager® and ProduXion Manager® from Aries Systems Corporation

Click here to access/download;Manuscript;Manuscript plain text.docx

Lead ammunition residues in a hunted Australian grassland bird, the stubble quail (*Coturnix pectoralis*): implications for human and wildlife health

Jordan O. Hampton[1,2¶*], Heath Dunstan[3¶], Simon D. Toop[3&], Jason S. Flesch[3&], Alessandro Andreotti[4&], Deborah J. Pain[5&]

[1] Faculty of Veterinary and Agricultural Sciences, University of Melbourne, Parkville, Victoria 3052, Australia

[2] Harry Butler Institute, Murdoch University, 90 South St, Murdoch WA 6150, Australia

[3] Game Management Authority, level 2, 535 Bourke Street, Melbourne, Victoria 3000, Australia

[4] Area Avifauna Migratrice, Istituto Superiore per la Protezione e la Ricerca Ambientale, Via Ca' Fornacetta 9, Ozzano Emilia 40064, Italy

[5] Department of Zoology, University of Cambridge, David Attenborough Building, Pembroke Street, Cambridge CB2 3QZ, United Kingdom

Corresponding author

* Email: Jordan.hampton@unimelb.edu.au (JOH)

[¶]These authors contributed equally to this work.

[&]These authors contributed equally to this work.

Abstract

Scavenging and predatory wildlife can ingest lead from ammunition and become poisoned when feeding on injured, or killed but unretrieved, game animals. Humans can similarly be exposed to ammunition-derived lead when consuming wild-shot game animals. Many studies have been performed to assess the degree of lead contamination in the carcasses of game animals to inform this risk. This scrutiny has not so far extended to Australia. Stubble quail (*Coturnix pectoralis*) is the only native non-waterfowl bird species that can be legally hunted in Victoria, Australia, and is commonly hunted with lead shot. The aim of this study was to characterize lead contamination in harvested stubble quail. The frequency, dimensions, and number of lead fragments embedded in carcasses were assessed through use of radiography (X-ray). From these data, the average quantity of lead available to scavenging wildlife was estimated along with potential risks to human consumers. We radiographed 37 stubble quail killed by hunters using lead shot (#9, 28 g) from 12-gauge shotguns in western Victoria, Australia, in Autumn 2021. Radiographs revealed that 81% of carcasses contained embedded pellets and/or fragments. We counted 60 shotgun pellets in 24 carcasses. In 14 birds, we detected both whole pellets and lead fragments; in 10, only pellets; in 6, we found only small fragments; and in 7, we found neither pellets nor fragments. On average, 1.62 embedded pellets were detected per bird. By excising and weighing a sample of 30 shotgun pellets (all had a mass of ~0.75 grain or 48.6 mg), we calculated an average lead load of 78 mg/100 g of body mass. This was a conservative estimate, because fragments were not considered. This level of lead contamination was comparable to hunted bird species examined using similar methods in Europe. The quantity and characteristics of lead ammunition residues found suggest that predatory and scavenging wildlife feeding on Australian stubble quail will be at risk of negative health impacts: health risks are also likely to extend to some groups of human consumers.

Introduction

Lead (Pb) is a toxic non-essential metal that affects multiple body systems in vertebrates, especially the nervous system. No levels of exposure are considered to be safe to humans [1], and a similar situation is likely to exist for other vertebrates. Dietary exposure to lead of ammunition origin poses health risks to humans [2], scavenging wildlife [3], and wild birds including wildfowl that ingest grit (gastroliths) [4]. There is currently considerable global focus on the health and environmental risks resulting from the use of lead ammunition in hunting, and its replacement with non-toxic alternatives is increasingly being mandated or considered across the world [5], especially in Europe [6], North America [7] and Japan [8].

Of conservation significance, the poisoning of wild birds following the ingestion of spent lead ammunition has been recognized for many decades, and in waterbirds and terrestrial gamebirds for over a century [9-11]. Also, many species of predatory and scavenging birds are affected across the world [3, 4]. Raptors are particularly exposed to lead poisoning due to the low pH values in their gizzard (1.2–1.4) that facilitates the absorption of metallic lead [12]. Long-lived species with a delayed sexual maturity and a low reproductive rate, such as eagles and vultures, suffer mostly because juveniles may accumulate a relevant burden of ammunition-derived lead before they start breeding [13], with possible adverse effects on their demography [14]. Indeed, the critically endangered California condor (*Gymnogyps californianus*) was driven to near-extinction as a result of lead poisoning [15].

Of public health significance, numerous studies have revealed that humans can ingest elevated levels of dietary lead while consuming game animals killed by lead ammunition [2, 16]. Ingested ammunition-derived lead is biologically available [17]: blood lead levels in people that frequently eat game shot with lead ammunition are generally higher than in people that do not, with levels increasing with the amount of game consumed [18, 19]. Those

groups of people most at risk from health impacts following exposure to ammunition-derived dietary lead include frequent consumers of game, and groups especially vulnerable to the effects of lead, including pregnant women and children. The high vulnerability of children results from them absorbing a higher proportion of dietary lead than adults, and the irreversible effects of lead on the developing nervous system. Reduced Intelligent Quotient (IQ) associated with such damage has been identified as a critical health endpoint for lead when assessing risks to children [20].

Lead of ammunition origin can be readily ingested and absorbed from game meat for two main reasons. Firstly, bullets and pellets tend to fragment in the body of game animals, producing numerous particles too small to be detected and removed during food preparation and mastication [17, 21]. Secondly, cooking processes may facilitate the solubilization of lead particles in the flesh, and this may be enhanced by certain processes, for example in the presence of acid ingredients (wine, vinegar or marinades) [22-24].

Given the importance of this issue, many studies have documented levels of lead contamination of wild-shot game meat. These include studies assessing the frequency, dimension, and number of lead fragments embedded in the carcasses of ungulates, waterbirds and ‘upland’ game birds to evaluate the risk related to the consumption of game meat [17, 21]. Despite the popularity of hunting in Australia, previously no data have been available on game birds hunted on this continent [25].

When compared to European practices, Australian recreational hunting is ~~relatively~~ poorly described in scientific literature [26]. Stubble quail (*Coturnix pectoralis*) is the only native grassland bird that can be legally hunted in Victoria, Australia. Stubble quail are a small (~100 g) ground-dwelling galliforms that exhibit nomadic behaviour, being capable of long-distance dispersal [27]. They display boom-and-bust abundance cycles [26] with a regular spring/early-summer breeding period and frequent second breeding known to occur in

late-summer/autumn if conditions are favourable [28-30]. They are commonly harvested in their tens or hundreds of thousands using shotguns and gundogs on privately owned agricultural land where cropping is the primary land use (Fig 1) and have been hunted in this way for over 100 years [31].

Currently, approximately 200,000 stubble quail are harvested each year in Victoria by approximately 28,000 licenced hunters [26], all are destined for human consumption, and most are hunted with lead shot. While lead shot has been banned for waterfowl hunting in many (but not all) Australian jurisdictions, its use continues to be permitted for non-waterbirds including grassland birds [25], notably the stubble quail. A 2020 survey of Victorian game licence holders revealed that an estimated 81% (95% CI = 76–84%) of stubble quail hunters still use lead shot [32].

As all harvested and retrieved stubble quail are consumed by humans, the continued use of lead gunshot may present a health risk to consumers, especially frequent consumers and vulnerable groups. An unknown number of shot and unretrieved stubble quail is also eaten by scavenging or predatory raptors, with potential negative effects on their population dynamic [14]. In spite of the long-term popularity of hunting stubble quail in southeastern Australia, no data relating to lead contamination have previously been available for this species. Here, we assessed lead contamination of the carcasses of stubble quail harvested by recreational hunters in Victoria using lead shot. This is the first description of lead contamination of Australian grassland game birds harvested with lead-based ammunition.

Materials and methods

Hunting methods

To produce results comparable to those of published international studies, we largely followed the methods employed in published studies of European game birds [33, 34]. Our study used hunter-collected birds harvested with lead shot (#9 (2.0 mm diameter), 28 g) from 12-gauge shotguns on private property in western Victoria, Australia (Fig 1) in Autumn (April–June: the legally allowed hunting season), 2021 [35]. Hunting with shotguns is the only technique permitted for recreational hunting of stubble quail in Victoria [32]. No birds were expressly killed for this study.

Fig 1. Typical methods used for recreational hunting of stubble quail (*Coturnix pectoralis*) in south-eastern Australia: shotgun with lead shot. This image shows the size of the birds relative to a firearm and a typical harvest for one hunter in one day: 20 birds. Photo: Heath Dunstan (HD).

Radiography

Harvested birds were immediately labelled with unique numbered metal tags (Fig 2), placed in zip-lock plastic bags and frozen at -20 °C. Birds were thawed and radiographed in a lateral position using a portable veterinary radiography unit (Cuattro, Golden, US) [36] set at 68 kVp and 1.0 mAs. From radiographs, the number of embedded pellets, their anatomical distribution, and the number and size of fragments [37] and their anatomical distribution were recorded (Fig 2).

Fig 2. Embedded lead shot (large bright white objects) and fragmentation centers (red circles) detected via radiography in a harvested stubble quail (*Coturnix pectoralis*): three pellets and three fragmentation centers are visible in this bird.

To describe the distribution of lead pellets and fragments, we subdivided the body of each bird into six sectors so that the anatomical parts normally consumed by humans or scavenging wildlife could be easily treated separately: 1) head and neck, 2) wings, 3) humerus and pectoral girdle, 4) thorax, 5) abdomen, 6) femur and tibiotarsus, and 7) tarsus and metatarsus [33]. We regarded Sectors 3, 4, 5, and 6 as corresponding to edible parts, i.e., those most commonly eaten by humans and scavenging wildlife.

From radiographs, we counted the number of whole shot pellets embedded in each sector. Thereafter, we assessed the frequency of lead fragmentation, recording the number of 'fragmentation centers' (clusters of radiodense particles or single macrofragments >0.5 mm) [33]. 'Microfragments' (<0.5 mm) are not generally easily detectable to the naked eye on radiographs [38, 39], although this can vary with image resolution. The fragments were scored independently of their dimensions as follows: 0 = none visible; 1 = 1–2 macrofragments; 2 = 2–4 microfragments; and 3 = >4 fragments (regardless of their size). The average level of lead contamination (mg of lead/100 g of body weight) was then calculated [33].

To evaluate the quantity of the embedded lead and the proportion of the pellet mass fragmented into small particles, we excised 30 shotgun pellets from dissected shot quail. These pellets were washed, dried, and accurately weighed by means of a Hornady® G3-1500 electronic scale (accuracy d = 0.1 grain) (Hornady, Grand Island, US).

Statistics

Descriptive statistics were used to assess the frequency and distribution of lead pellets and fragments in examined stubble quail.

Results

We examined 37 stubble quail. Our sample size was limited by the donations of participating recreational hunters. The mean weight of the examined stubble quail was 101 g (SD = 12, range = 74–122, n = 37). Ammunition residues were found in the majority of carcasses: radiographs revealed that 30 birds (81.1%) contained either embedded pellets and/or fragments and 28 of these birds (75.7%) contained these lead residues in the anatomical regions most commonly eaten by scavenging wildlife or humans. In 14 birds (37.8%), we detected both whole pellets and lead fragments; in 10 (27.0%) only pellets; in 6 (16.2%) we found only small fragments; and in 7 (18.9%) we found neither pellets nor fragments. Pellets and fragments were embedded in all body sectors.

Shotgun pellets

Radiographs (Fig 2) revealed 60 pellets in 24 stubble quail (mean = 1.62, SD = 1.67, range = 0–5, n = 37). Most of the pellets (65.6 %; n = 51) were located in the edible sectors (3–6) with the remaining 9 pellets (15%) found in the head/neck, with no pellets detected in the wings or distal parts of legs (Table 1). All shotgun pellets excised from dissected birds (n = 30) had a mass of ~0.75 grain or 48.6 mg).

Table 1 Distribution of lead fragment classes in different body sectors. Fragment scores: 0 = none visible; 1 = 1–2 macrofragments; 2 = 2–4 microfragments; 3 = >4 fragments [33].

**Score**	**Head/ neck**	**Wings**	**Humerus / pectoral girdle**	**Thorax**	**Abdomen**	**Femur / tibiotarsus**	**Tarsus / metatarsus**	**TOTAL**
Score 1	1	0	2	0	2	2	2	9
Score 2	0	2	2	0	2	2	2	10
Score 3	1	3	4	2	2	5	2	19

Fragments

A minimum of 38 fragmentation centers (Fig 2) were detected in 26 stubble quail (mean = 1.0, SD = 1.3, range = 0–5, *n* = 37). Most of the fragmentation centers (*n* = 25; 65.8 %) were located in the edible sectors (3–6) with smaller fractions found in the distal parts of legs (*n* = 6; 15.8%), wings (*n* = 5; 13.2%), and head/neck (*n* = 2; 5.3%). (Table 1). In 19 cases (50%) of the fragments were assigned to Class 3 (>4 fragments), in 10 (26.3%) were Class 2 and in 9 (23.7%) were Class 1.

Lead load

We calculated an average number of embedded pellets per unit of body mass (1.61/100 g of body weight) and resultant lead load of 78.2 mg/100 g of body mass. This lead mass was similar in stubble quail to most hunted bird species examined using similar methods in Europe (Table 2). This estimate can be regarded as conservative because fragments were not considered.

Table 2 Lead contamination in stubble quail (*Coturnix pectoralis*) from south-eastern Australia in comparison to published results from European game bird species. This table is updated from a past study [33]. Mean body mass and pellet sizes were taken from standard ornithological texts and from hunting sources, respectively.

Game bird species	*n* birds	*n* pellets/ bird	Reference for *n* of shot	Body mass (g)	Reference for mass	*n* pellets /100 g	Shot size	Shot mass per pellet (mg)	Pb mass (mg)/100 g
Stubble quail (*Coturnix pectoralis*)	37	1.62	This study	101	This study	1.61	9	48.6	78.2
European starlings (*Sturnus vulgaris*)	196	0.65	[33]	70	[40]	0.93	10–11	29.1	27
Red-legged partridge (*Alectoris rufa*)	64	3.67	[23]	500	[41]	0.73	5–6	130.1	95
Thick-billed murre (*Uria lomvia*)	50	3.7	[42]	900	[43]	0.41	4	175.6	72
Common eider (*Somateria mollissima*)	25	10.40	[44]	2000	[45]	0.52	0–6	219.2	114
Eurasian woodcock (*Scolopax rusticola*)	59	3.64	[34]	304	[34]	1.20	7.5–12	37.3	45−52

Discussion

To our knowledge, this is the first study to report embedded lead contamination levels in a harvested Australian game bird. The embedded lead load in harvested stubble quail was comparable to most European game bird species studied (Table 2). This is also ~~broadly~~ similar to results from the UK, where radiography was carried out on 121 oven-ready gamebirds of 6 species [21]. Even though the examined birds were without feathers, viscera and head, 65% of them contained at least one large fragment (estimated to be at least the size of half a pellet) or whole pellet. The mean number of shot/large fragment per bird ranged from 0.95–3.32 with an overall mean of 2.17 pellets per bird.

In game carcasses, lead particles may derive from both lethal shot and old wounds that have subsequently healed. In some game species, the proportion of apparently healthy birds with embedded pellets may be unexpectedly high (often around 25% in wildfowl) [46] and has been reported to increase with age [47]. In apparently healthy birds, larger Australian duck species tended to contain proportionally more pellets than smaller species [47]. Similarly, a UK study found that the number of shotgun pellets embedded in six species of dead gamebirds was significantly positively correlated with body size [21]. Apparently healthy stubble quail, with a weight of approximately 100 g, are unlikely to carry as high a proportion of embedded shot (from surviving previous shooting) as the commonly shot duck species in Australia, as these ducks have an average body weight about 7 times greater [48]. This conjecture is also supported by a European study that did not find a higher prevalence of embedded shot in adult Eurasian woodcock (*Scolopax rusticola*) than in juveniles (mean weight around 300 g) [34]. Game ducks in south-eastern Australia are harvested using non-toxic (usually steel) shot which is ballistically different to lead, but rates of shot retention (embedded shot) are likely to be similar [49]. Nonetheless, a small proportion of embedded shot found in stubble quail may have been carried from previous shooting seasons.

Risks to human health

The weight of a game meal is generally considered to range from about 100–200 g of meat per person [2, 18, 21, 42]. Assuming that about half of the weight of a stubble quail is edible meat, a single portion for an adult consumer would comprise approximately 2–4 oven-ready stubble quail. In practice, it is more common for people to consume 4–6 stubble quail in a single meal (H. Dunstan, S. Toop, personal communication). Our study reveals a high probability that at least one bird in a meal could be contaminated by whole pellets or visible fragments; more are likely to contain tiny fragments. Most whole pellets or large fragments will probably be removed during food preparation or at the table, although occasionally pellets may be inadvertently ingested and some associated lead solubilized. Due to the small surface area to volume ratio of lead pellets and large fragments, only a small proportion of the lead mass is likely to be absorbed into the bloodstream. Rare exceptions include cases where lead shot or bullets are retained in the intestinal tract resulting in elevated blood lead and/or appendicitis [50-52]. However, two factors mean that the risks are higher than this would otherwise imply. These are the presence of very small fragments that cannot readily be removed during food preparation [53], and the solubilization of lead from pellets during the cooking process. Consequently, health risks from consuming stubble quail will be related to lead concentrations in the meat after removal of whole shot and large fragments, and the frequency of consumption of stubble quail over time.

During the passage of shot through gamebirds, many micro-fragments are widely distributed throughout the flesh and tissue lead concentrations are frequently highly elevated. High lead concentrations have been found in all meals prepared from gamebirds found by X-ray to contain many lead shot, and also in some meals prepared from birds with few or no shot visible on X-rays [21]. This probably resulted from the presence of tiny radio-dense

fragments, (presumably lead), seen on X-rays of the carcasses of 76% of the 121 birds analysed, including most of those in which all gunshot had passed through the body. Most such fragments were very tiny (i.e. less than about a tenth of a shot in size) and too small and scattered to be detected or removed by a consumer.

The coarse resolution of two-dimensional radiographic studies makes it difficult to identify lead fragments smaller than about 0.1 mm [2] and other authors have suggested that unambiguous identification of lead fragments is hampered at sizes below 0.4 mm or 0.5 mm [38, 39]. However, many lead particles in animals shot with lead ammunition may be considerably smaller than this. Inductively coupled plasma-mass spectrometry in single-particle mode (spICP-MS) has been applied to meat from wild boar/feral pigs (*Sus scrofa*) and roe deer (*Capreolus capreolus*) [53] and has found numerous lead particles ranging from 40 to 750 nanometres (nm) in size within 10 cm of the bullet wound channel. Even the largest of these, at 750 nm, were over 100 times smaller than the smallest particle detectable by radiography.

The implications of studies on ammunition fragmentation are that even if whole shot and large lead fragments in stubble quail and other game species are removed at the table, numerous tiny lead fragments are likely to remain in the majority of birds. These are likely to be more readily solubilized and absorbed than whole shot due to their larger surface area/volume ratio. This is supported by studies that have observed that the concentration of bioaccessibile lead in cooked red-legged partridge (*Alectoris rufa*) meat is significantly related to the presence of X-ray visible pellets and/or fragments, irrespective of their dimension and number [23]. Similarly, previous studies have demonstrated that lead contamination in game meat is more closely associated with the presence of small particles scattered throughout tissues, rather than whole pellets [42, 54].

Past studies have found that both the number of shotgun pellets/large fragments (which were removed after cooking gamebirds and before analysis for lead) and the number of small fragments (which were not removed) have significant separate effects on lead concentration in a cooked gamebird meal [21]. This suggests that lead in game meat meals is solubilized from both whole/large pellets during the cooking process and from small fragments. Collectively these results suggest that the availability of lead from ammunition particles is strongly related to their surface area and not just their mass, since the larger surface areas of small particles facilitates lead solubilization during cooking or digestion. In addition to fragmentation, certain game storage and cooking methods may also influence the availability of lead in game meat meals [22-24].

One UK study found lead concentrations in edible gamebird tissue, after the removal of shot and large fragments, to be on average 1.181 ppm (wet weight) for six species of gamebird (*n* = 121), with no significant variation in tissue lead concentration across the species [21]. This mean level is approximately twelve times the Maximum Levels (MLs) permitted in domesticated meat (cattle, sheep, pigs and poultry) marketed in the EU (EC 2006) and listed in the Codex Alimentarius General Standard for Contaminants and Toxins (2019) for commodities moving in international trade [55]. While MLs for lead have not so far been set specifically for game meat within global standards or EU law, it has been recommended that such standards be set [56] and the profile of this issue is increasing internationally.

Although, as discussed, a greater mass of lead gunshot relative to body weight is generally found in large gamebird species rather than in small species like the stubble quail (Table 2) [21], this does not necessarily translate to the lead concentrations found in meat consumed by humans [21]. This is because tiny fragments left by the passage of lead projectiles may be responsible for much of the final lead loads to which human consumers

will be exposed. In six UK gamebird species, while body size was positively and significantly related to the number of retained shot, lead concentrations in gamebird meals, analysed after removal of shot and large fragments, thus simulating realistic human exposure, did not vary significantly between species [21].

Therefore, given both the frequency of whole pellets and fragments recorded in stubble quail it can be inferred that the people who eat stubble quail in Australia are likely to be comparably exposed to lead to those consuming birds shot with lead ammunition in Europe, including partridges, seabirds and songbirds. The elevated lead levels found in gamebirds shot with lead ammunition are of concern for the health of human consumers, especially those who consume such meat frequently, or are especially vulnerable to the effects of lead, such as children and pregnant women [2]. Additional work analysing lead concentrations in the edible meat of stubble quail following the removal of readily visible ammunition particles, to simulate realistic human exposure, would further confirm this.

Risks to scavenging and predatory birds

In addition to human health risks, considerable risks exists for scavenging and predatory raptors that feed on shot game species that are wounded or killed but not recovered by the hunter [4]. The lead loads in Table 2 are more directly relevant to scavengers and predators than humans. In contrast to human consumers, scavenging and predatory wildlife will ingest whole shotgun pellets along with fragments [57]. Raptors ingesting lead shotgun pellets may eliminate them rapidly through defecation or regurgitation, or retain them for days or weeks and their highly acidic stomach conditions can result in considerable erosion/solubilization of ingested shot [58]. Consequently, the lead loads from shot found in stubble quail and other shot species (Table 2) along with the numerous lead fragments, together present considerable risks. Among raptors preying on stubble quail, the wedge-tailed

eagle (*Aquila audax*) is known to accumulate relevant amounts of lead in bone [59] as a result of the ingestion of lead-based ammunition [60]. Given that this eagle species is a K-strategist, with a high adult survival rate and a low productivity [61], lead poisoning probably exerts adverse effects also at the population level.

We conclude that the lead load in harvested stubble quail was similar to most game bird species studied in Europe. The quantity and characteristics of ammunition residues that were found suggest that game meat consumers, including humans and scavenging and predatory birds and other wildlife, are exposed to considerable quantities of lead. The use of lead shot for stubble quail hunting presents avoidable health risks to both people and wildlife which could be eliminated through a transition to lead-free shot as is increasingly happening in other jurisdictions and has already occurred in Victoria for game ducks.

Supporting information

S1 Data.

(CSV)

Acknowledgments

An observational Animal Ethics Committee (AEC) licence from Murdoch University (O3103/19) supported this study. We thank Daniel Airo-Farulla for assistance with sample collection. We thank the McKenzie Fellowship program of the University of Melbourne and the Victorian Game Management Authority for funding this study.

Author Contributions

Conceptualization: Jordan O. Hampton, Heath Dunstan.

Data curation: Jordan O. Hampton.

Formal analysis: Jordan O. Hampton, Alessandro Andreotti, Deborah J. Pain.

Funding acquisition: Simon D. Toop, Jordan O. Hampton.

Investigation: Heath Dunstan, Jordan O. Hampton.

Methodology: Jordan O. Hampton, Heath Dunstan, Alessandro Andreotti, Deborah J. Pain.

Project administration: Jordan O. Hampton.

Resources: Jordan O. Hampton, Simon D. Toop.

Software: Jordan O. Hampton.

Supervision: Jordan O. Hampton.

Validation: Jordan O. Hampton.

Visualization: Jordan O. Hampton.

Writing – original draft: Jordan O. Hampton.

Writing – review & editing: Jordan O. Hampton, Heath Dunstan, Simon D. Toop, Jason S. Flesch, Alessandro Andreotti, Deborah J. Pain.

References

1. World Health Organization. Lead poisoning facts sheet Geneva, Switzerland: World Health Organization; 2021. Available from: https://www.who.int/news-room/fact-sheets/detail/lead-poisoning-and-health.
2. Green RE, Pain DJ. Risks to human health from ammunition-derived lead in Europe. Ambio. 2019;48(9):954–68.
3. Monclús L, Shore RF, Krone O. Lead contamination in raptors in Europe: a systematic review and meta-analysis. Science of the Total Environment. 2020;748:141437.
4. Pain DJ, Mateo R, Green RE. Effects of lead from ammunition on birds and other wildlife: a review and update. Ambio. 2019;48(9):935–53.

5. Thomas VG, Kanstrup N, Pain DJ. Promoting the transition to non-lead hunting ammunition in the European Union through regulation and policy options. Environmental Policy and Law. 2021;51(4):239–54.

6. Gerofke A, Ulbig E, Martin A, Müller-Graf C, Selhorst T, Gremse C, et al. Lead content in wild game shot with lead or non-lead ammunition–Does “state of the art consumer health protection” require non-lead ammunition? PloS One. 2018;13(7):e0200792.

7. Schulz JH, Stanis SAW, Hall DM, Webb EB. Until It's a regulation It's not my fight: Complexities of a voluntary nonlead hunting ammunition program. Journal of Environmental Management. 2021;277:111438.

8. Ishii C, Ikenaka Y, Nakayama SM, Kuritani T, Nakagawa M, Saito K, et al. Current situation regarding lead exposure in birds in Japan (2015–2018); lead exposure is still occurring. Journal of Veterinary Medical Science. 2020;82(8):1118–23.

9. Jordan JS, Bellrose FC. Lead poisoning in wild waterfowl. llinois Natural History Survey Biological Notes. 1951;26:1–27.

10. Calvert H. Pheasants poisoned by swallowing shots. The Field. 1876;47:189.

11. Wetmore A. Lead poisoning in waterfowl. Washington, D.C., USA: US Department of Agriculture; 1919.

12. Krone O. Lead poisoning in birds of prey. In: Sarasola JH, Grande JM, Negro JJ, editors. Birds of Prey. New York City, USA: Springer; 2018. p. 251–72.

13. Bassi E, Facoetti R, Ferloni M, Pastorino A, Bianchi A, Fedrizzi G, et al. Lead contamination in tissues of large avian scavengers in south-central Europe. Science of The Total Environment. 2021;778:146130.

14. Gil-Sánchez JM, Molleda S, Sánchez-Zapata JA, Bautista J, Navas I, Godinho R, et al. From sport hunting to breeding success: patterns of lead ammunition ingestion and its effects on an endangered raptor. Science of the Total Environment. 2018;613:483–91.

15. Finkelstein ME, Doak DF, George D, Burnett J, Brandt J, Church M, et al. Lead poisoning and the deceptive recovery of the critically endangered California condor. Proceedings of the National Academy of Sciences of the United States of America. 2012;109(28):11449–54.

16. Knutsen HK, Brantsæter A-L, Alexander J, Meltzer HM, editors. Associations between consumption of large game animals and blood lead levels in humans in Europe: the Norwegian experience. Oxford Lead Symposium; 2014; Oxford, UK: University of Oxford.

17. Hunt WG, Watson RT, Oaks JL, Parish CN, Burnham KK, Tucker RL, et al. Lead bullet fragments in venison from rifle-killed deer: potential for human dietary exposure. PloS One. 2009;4(4):e5330.

18. Green R, Pain D. Potential health risks to adults and children in the UK from exposure to dietary lead in gamebirds shot with lead ammunition. Food and Chemical Toxicology. 2012;50(11):4180–90.

19. Iqbal S, Blumenthal W, Kennedy C, Yip FY, Pickard S, Flanders WD, et al. Hunting with lead: association between blood lead levels and wild game consumption. Environmental Research. 2009;109(8):952–9.

20. EFSA. Scientific opinion on lead in food. EFSA Panel on Contaminants in the Food Chain (CONTAM). EFSA Journal. 2010;8(4):1570.

21. Pain DJ, Cromie RL, Newth J, Brown MJ, Crutcher E, Hardman P, et al. Potential hazard to human health from exposure to fragments of lead bullets and shot in the tissues of game animals. PloS One. 2010;5(4):e10315.

22. Mateo R, Rodriguez-De La Cruz M, Vidal D, Reglero M, Camarero P. Transfer of lead from shot pellets to game meat during cooking. Science of the Total Environment. 2007;372(2–3):480–5.

23. Mateo R, Baos AR, Vidal D, Camarero PR, Martinez-Haro M, Taggart MA. Bioaccessibility of Pb from ammunition in game meat is affected by cooking treatment. PLoS One. 2011;6(1):e15892.

24. Schulz K, Brenneis F, Winterhalter R, Spolders M, Fromme H, Dietrich S, et al. Marination increases the bioavailability of lead in game meat shot with lead ammunition. Journal of Nutritional Science. 2021;10:e24.

25. Hampton JO, Laidlaw M, Buenz E, Arnemo JM. Heads in the sand: public health and ecological risks of lead-based bullets for wildlife shooting in Australia. Wildlife Research. 2018;45:287–306.

26. Moloney PD, Gormley AM, Toop SD, Flesch JS, Forsyth DM, Ramsey DSL, et al. Contrasting long-term trends in the harvest of native and introduced species by recreational hunters in Australia revealed using Bayesian modelling. Wildlife Research. In press.

27. Frith HJ, Waterman MH. Movements of stubble quail, *Coturnix pectoralis*, from South Australian grainfields. Wildlife Research. 1977;4(1):85–90.

28. Toop SD. Reproductive timing and habitat preference in the stubble quail. Honours Thesis, University of Ballarat; 1994.

29. Cruise J. Stubble quail, *Coturnix pectoralis*, and their breeding behaviour. Emu-Austral Ornithology. 1966;66(1):39–45.

30. Frith H, Carpenter SJ. Breeding of the stubble quail, *Coturnix pectoralis*, in south-eastern Australia. Wildlife Research. 1980;7(1):117–37.

31. Kinghorn J. Economic value of the stubble quail. Emu-Austral Ornithology. 1926;26(2):112–9.

32. Game Management Authority. GMA hunting methods survey 2020. Melbourne, Australia: Game Management Authority, 2020.

33. Andreotti A, Borghesi F. Embedded lead shot in European starlings *Sturnus vulgaris*: an underestimated hazard for humans and birds of prey. European Journal of Wildlife Research. 2013;59(5):705−12.

34. Andreotti A, Borghesi F, Aradis A. Lead ammunition residues in the meat of hunted woodcock: A potential health risk to consumers. Italian Journal of Animal Science. 2016;15(1):22–9.

35. Moloney P, Hampton J. Estimates of duck and Stubble Quail harvests in Victoria for 2020: results from surveys of Victorian Game Licence holders in 2020. Melbourne, Australia: Game Management Authority, 2020.

36. Hampton JO, Eccles G, Hunt R, Bengsen AJ, Perry AL, Parker S, et al. A comparison of fragmenting lead-based and lead-free bullets for aerial shooting of wild pigs. Plos One. 2021;16(3):e0247785.

37. Herring G, Eagles-Smith CA, Wagner MT. Ground squirrel shooting and potential lead exposure in breeding avian scavengers. PLoS One. 2016;11(12):e0167926.

38. Hunt WG, Burnham W, Parish CN, Burnham KK, Mutch B, Oaks JL. Bullet fragments in deer remains: implications for lead exposure in avian scavengers. Wildlife Society Bulletin. 2006;34(1):167–70.

39. Knott J, Gilbert J, Hoccom DG, Green RE. Implications for wildlife and humans of dietary exposure to lead from fragments of lead rifle bullets in deer shot in the UK. Science of the Total Environment. 2010;409(1):95–9.

40. Spina F, Licheri D. Biodiversità dell'avifauna italiana: variabilità morfologica nei Passeriformi: parte III: (*Muscicapidae-Emberizidae*). Biol Cons Fauna. 2003;13:1–177.

41. Cramp S. Handbook of the birds of Europe, the Middle East and North Africa: the birds of the Western Palearctic. Volume II: hawks to bustards. Oxford, UK: Oxford University Press; 1980.

42. Johansen P, Asmund G, Riget F. Lead contamination of seabirds harvested with lead shot—implications to human diet in Greenland. Environmental Pollution. 2001;112(3):501–4.

43. Cramp S. Handbook of the birds of Europe, the Middle East and North Africa: the birds of the Western Palearctic. Volume IV: terns to woodpeckers. Oxford, UK: Oxford University Press; 1989.

44. Johansen P, Asmund G, Riget F. High human exposure to lead through consumption of birds hunted with lead shot. Environmental Pollution. 2004;127(1):125–9.

45. Cramp S, Simmons K. Birds of Europe the Middle East and North Africa: the birds of the Western Palearctic. Volume I: ostrich to ducks. Oxford, UK: Oxford University Press; 1977.

46. Pain DJ, Cromie R, Green RE. Poisoning of birds and other wildlife from ammunition-derived lead in the UK. In: Delahay RJ, Spray CJ, editors. Oxford Lead Symposium; Edward Grey Institute, UK: University of Oxford; 2014. p. 58–84.

47. Norman F. The incidence of lead shotgun pellets in waterfowl (Anatidae and Rallidae) examined in South-Eastern Australia between 1957 and 1973. Wildlife Research. 1976;3(1):61–71.

48. Del Hoyo J, Del Hoyo J, Elliott A, Sargatal J. Handbook of the birds of the world. Volume 1: ostrich to ducks. Barcelona, Spain: Lynx Edicions; 1992.

49. Pierce BL, Roster TA, Frisbie MC, Mason CD, Roberson JA. A comparison of lead and steel shot loads for harvesting mourning doves. Wildlife Society Bulletin. 2015;39(1):103–15.

50. Ozkan OV, Muderris V, Altintoprak F, Yagmurkaya O, Yalkin O, Celebi F. An unusual cause of abdominal pain: three lead pellets within the appendix vermiformis. Case Reports in Surgery. 2015;2015:496372.

51. Khazindar AR, Thabit RA, Badeeb A. Bullet appendicitis: an unusual cause to a rather straight-forward diagnosis. Cureus. 2021;13(7):e16638.

52. Rosenberg AG, Haynes JH. Management of ingested lead pellets with elevated blood lead levels in a pediatric patient. Journal of Pediatric Gastroenterology and Nutrition. 2019;69(6):e159–e60.

53. Kollander B, Widemo F, Ågren E, Larsen EH, Loeschner K. Detection of lead nanoparticles in game meat by single particle ICP-MS following use of lead-containing bullets. Analytical and Bioanalytical Chemistry. 2017;409(7):1877–85.

54. Scheuhammer A, Perrault J, Routhier E, Braune B, Campbell G. Elevated lead concentrations in edible portions of game birds harvested with lead shot. Environmental Pollution. 1998;102(2–3):251–7.

55. Food and Agriculture Organization of the United Nations. Codex Alimentarius international food standards: General standard for contaminants and toxins in food and feed Rome, Italy: Food and Agriculture Organization of the United Nations, World Health Organization, 2019. Available from: https://www.awe.gov.au/agriculture-land/farm-food-drought/food/codex.

56. Thomas VG, Pain DJ, Kanstrup N, Green RE. Setting maximum levels for lead in game meat in EC regulations: An adjunct to replacement of lead ammunition. Ambio. 2020;48(9):954–68.

57. Kendall RJ, Lacker Jr TE, Bunck C, Daniel B, Driver C, Grue CE, et al. An ecological risk assessment of lead shot exposure in non- waterfowl avian species: upland game birds and raptors. Environmental Toxicology and Chemistry. 1996;15(1):4–20.

58. Golden NH, Warner SE, Coffey MJ. A review and assessment of spent lead ammunition and its exposure and effects to scavenging birds in the United States. In: de

Voogt P, editor. Reviews of Environmental Contamination and Toxicology. 237. Cham, Switzerland: Springer International; 2016. p. 123–91.

59. Hampton JO, Specht AJ, Pay JM, Pokras M, Bengsen AJ. Portable X-ray fluorescence for bone lead measurements of Australian eagles. Science of the Total Environment. 2021;789:147998.

60. Lohr MT, Hampton JO, Cherriman S, Busetti F, Lohr C. Completing a worldwide picture: preliminary evidence of lead exposure in a scavenging bird from mainland Australia. Science of The Total Environment. 2020;715:135913.

61. Olsen P. Wedge-tailed eagle. Collingwood, Australia: CSIRO Publishing; 2005.

Figure

Click here to access/download;Figure;Fig 1.tiff

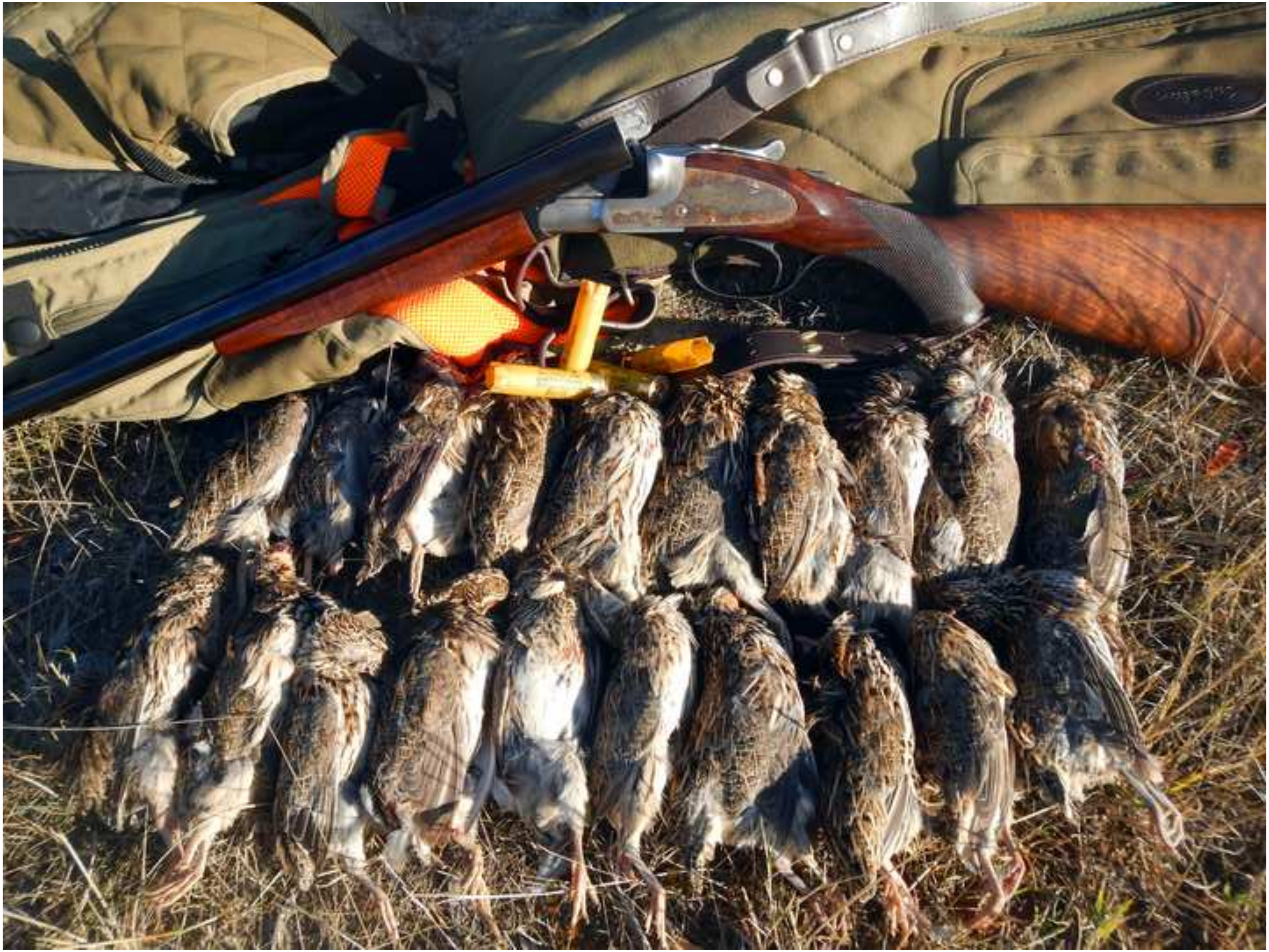

Figure

Click here to access/download;Figure;Fig 2.tiff

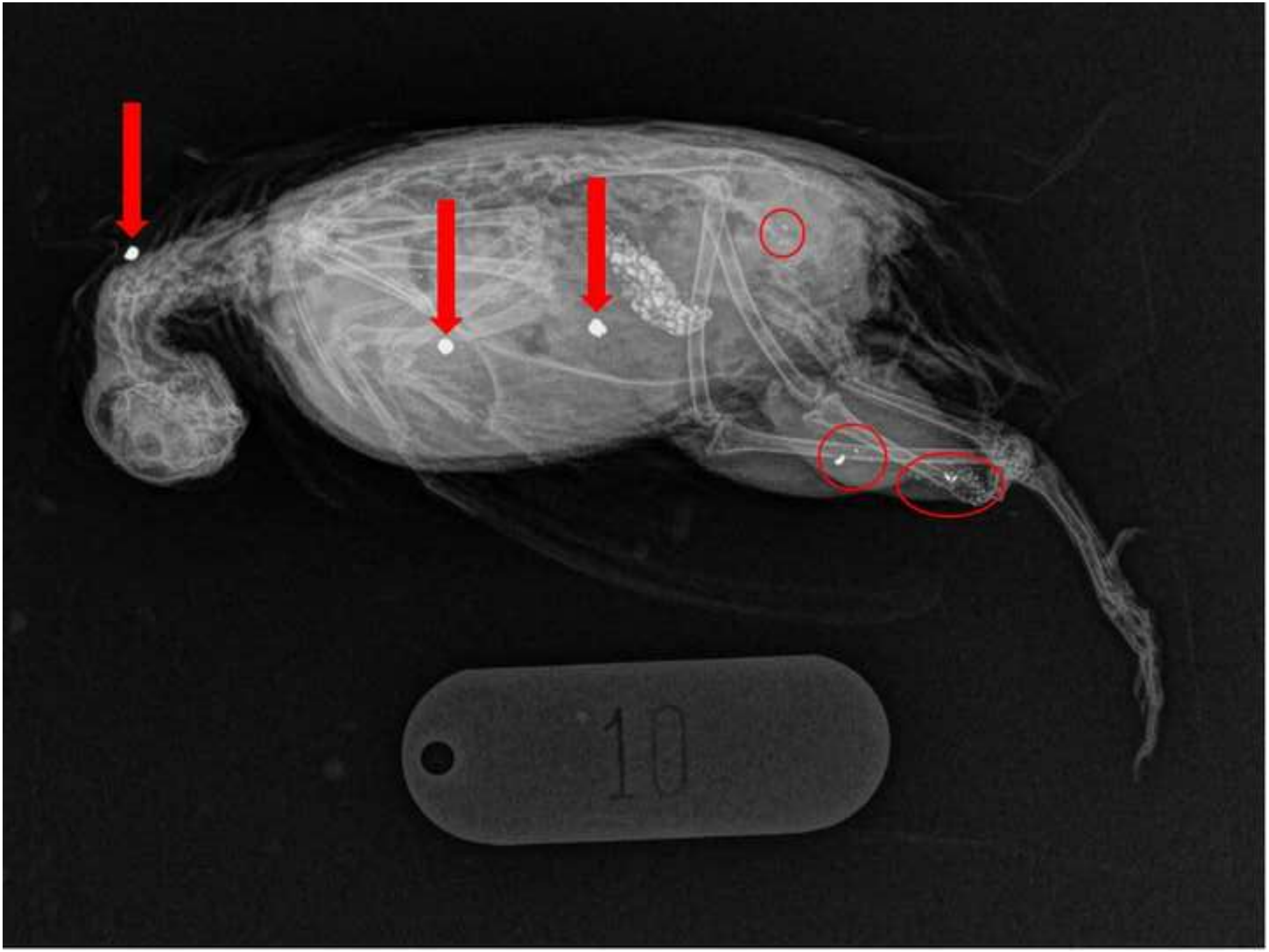

Click here to access/download
Supporting Information
S1.csv
